# Identification of a Six Gene Prognosis Signature for Papillary Thyroid Cancer Using Multi-Omics Methods and Bioinformatics Analysis

**DOI:** 10.3389/fonc.2021.624421

**Published:** 2021-03-18

**Authors:** He Ren, Xin Liu, Fuxin Li, Xianghui He, Na Zhao

**Affiliations:** Department of General Surgery, Tianjin Medical University General Hospital, Tianjin Medical University, Tianjin, China

**Keywords:** papillary thyroid cancer, hub genes, signaling pathways, bioinformatics, tumor infiltrating

## Abstract

Papillary thyroid carcinoma (PTC) is the most common subtype of thyroid cancer. PTC is typically curable with an excellent survival rate; however, some patients experience disease recurrence or death. This study aimed to discover potential key genes and signaling pathways of PTC, which could provide new insights for thyroid lesions. Four GEO microarray datasets were integrated to screen for candidate genes involved in PTC progression. A total of 164 upregulated and 168 downregulated differentially expressed genes (DEGs) were screened. Gene Ontology/Kyoto Encyclopedia of Genes and Genomes were used in pathway enrichment analyses for DEGs. A protein-protein interaction network was then built and analyzed utilizing STRING and Cytoscape, followed by the identification of 13 hub genes by cytoHubba. *CDH3, CTGF, CYR61, OGN, FGF13*, and *CHRDL1* were selected through survival analyses. Furthermore, immune infiltration, mutations and methylation analysis indicated that these six hub genes played vital roles in immune surveillance and tumor progression. ROC and K-M plots showed that these genes had good prognostic values for PTC which was validated by TCGA dataset. Finally, GSEA for a single hub gene revealed that each candidate hub gene had close associations with PTC development. These findings provided new insights into PTC pathogenesis and identified six candidate gene prognosis signature for PTC.

## Introduction

Thyroid cancer (THCA) is one of the most common endocrine malignancies worldwide, and has an increasing incidence (>3% per year) (1). In 2018, the global incidence of THCA in women was 10.2 per 100,000 (3× that of men), and the disease accounted for 5.1% (1/20 cancer diagnoses) of all cancers in women (2). Thyroid cancer is the sixth most common type of cancer in American women, with approximately 37,810 new cases diagnosed and 1,150 deaths in 2019 (1). Among Chinese women, THCA is the most commonly diagnosed cancer in women before the age of 30, and 67,900 new cases and 4,300 deaths occurred in 2015 (3). THCA can be subdivided into multiple subtypes. The most common subtype is papillary thyroid cancer (PTC), accounting for about 80% of all THCAs (4). Although the majority of patients with PTC have a good prognosis, >10% of patients experience disease recurrence or metastasis during long-term follow-up (5). Therefore, it is necessary to elucidate the pathogenesis of PTC and identify effective prognostic biomarkers.

In recent years, *BRAF* and *RAS* point mutations have been shown to closely precede the development of PTC (6, 7). In addition, rearrangements involving *RET* and *NTRK1* lead to activation of the mitogen-activated protein kinase (MAPK) signaling pathway, which can accelerate PTC progression (8, 9). Despite significant research advances, the underlying mechanisms of PTC tumorigenesis remain elusive. Therefore, more relevant genes need to be identified to fully understand the mechanism of PTC progression. The widespread application of next-generation sequencing and other gene microarray technologies have resulted in the generation of numerous public databases that store a high number of large functional genome datasets. The rapid development of computational tools has provided another key to identifying potential genes and pathways involved in PTC (10). The heterogeneity between study samples and studies from a single cohort often pose challenges with data interpretation (11, 12). The data are often incomplete or inconsistent with one another. As a result, a comprehensive analysis of integrated gene expression data using bioinformatics methods can address this shortcoming.

In the present study, raw data from four microarray datasets from the Gene Expression Omnibus (GEO; http://www.ncbi.nlm.nih.gov/geo/) were downloaded and used for bioinformatics analyses. A six gene signature was identified by combining the specific gene expression comparison, Gene Ontology (GO)/Kyoto Encyclopedia of Genes and Genomic Pathways (KEGG) analysis, protein-protein interaction (PPI) network construction, clinical-pathological features, infiltrating immune cell analysis, mutation, and methylation data. Finally, a gene set enrichment analysis (GSEA) was also used to investigate potential biological functions.

## Materials and Methods

### Data Preparation and Pre-Processing

Five raw PTC-associated gene expression datasets, including GSE3678, GSE6004, GSE33630, GSE53157, and GSE58545, were downloaded from NCBI-GEO (https://www.ncbi.nlm.nih.gov/gds/). Among them, the GSE3678, GSE6004, GSE33630 and GSE53157 datasets were based on the GPL570 [HG-U133_Plus_2] Affymetrix Human Genome U133 Plus 2 Array platform. The GSE58545 dataset was analyzed on the GPL96 [HG-U133A] platform, which was used to test the correlation of screened hub genes. The raw data from these datasets were processed using the R language statistical software (version 3.6.1). The horizontal linear model, affy (13), and affyPLM packages (14) were used for the probe horizontal. Box plots of RNA degradation and Normalized Unscaled Standard Error (NUSE) were plotted to test the trend consistency of the data after the quality of the data was analyzed. RNA degradation was checked using the AffyRNAdeg function and microarray datasets with consistent trends and good RNA quality were selected for follow-up analyses. Data from The Cancer Genome Atlas (TCGA; https://cancergenome.nih.gov/)) was used as an external validation dataset.

### Differentially Expressed Gene Screening and Integration

To ensure comparability and completeness of the dataset, gene expression profiling data were standardized and batch effects were removed. Normalization was performed using the RMA function in the Affy package (13). Differentially expressed genes (DEGs) between the tumor and peri-tumor tissues were screened using the LIMMA package (15). Threshold values of log_2_FC>1 and adjusted P<0.05 were considered statistically significantly meaningful and the dataset (GSE3678, GSE6004, GSE33630, and GSE53157) DEGs were subjected to overlapping analyses using the website (http://bioinformatics.psb.ugent.be/beg/).

### Gene Ontology (GO) Enrichment and Kyoto Encyclopedia of Genes and Genomes (KEGG) Pathway Analysis

GO was used to make predictions based on the molecular function (MF), cellular components (CC), and biological processes (BP) of potential functions of target genes. KEGG enrichment analysis was used to determine the functional properties of DEGs. An online annotation, visualization, and integrated discovery database (16) (DAVID; http://david.abcc.ncifcrf.gov/) and Metascape (17) (http://metascape.org) were used to access GO and KEGG pathway enrichment analyses. P<0.05 was deemed statistically significant.

### PPI Network Construction and Hub Gene Identification

The Search Tool for the Retrieval of Interacting Genes/Proteins (STRING) database search tool is an online tool used to evaluate PPI information (18). In this study, DEGs with co-expression coefficients greater than 0.4 were extracted from the STRING database. The Cytoscape software, a publicly available bioinformatics software platform for visualizing and analyzing molecular interaction networks, was used to build a PPI network (19). Hub genes were selected from the intersection of the top 65 genes calculated using 12 topological analysis methods by using the Cytoscape plugin cytoHubba (20).

### The cBioPortal for Cancer Genomics Analysis

The cBioPortal for Cancer Genomics (http://www.cbioportal.org/) is an open source platform that supports visualization, analysis, and downloads of multiple cancer genomic datasets (21). The frequency of genetic alterations in 13 hub genes in patients with PTC and their relationship to survival outcomes were explored by using cBioPortal.

### Gene Expression Profiling Interactive Analysis Database Analysis

Gene Expression Profiling Interactive Analysis (GEPIA; http://gepia.cancer-pku.cn/) is a newly developed interactive web server for analyzing the RNA sequencing expression data of 9,736 tumors and 8,587 peri-tumor samples from The Cancer Genome Atlas (TCGA) and the Genotype-Tissue Expression (GTEx) databases using a standard processing pipeline (22). Disease-free survival (DFS) is the duration of time from the start of treatment to the date of progression, the start date of second-line treatment, or the date of death, whichever occurs first. OS is the length of time from the date of diagnosis or first treatment to the date of death or the last follow-up visit. We used GEPIA to compare gene expression and to evaluate the contribution of the screened candidate genes to OS and DFS of PTC patients.

### Clinical Patients’ Samples and Transcriptome Sequencing Validation

Tumor and paired peri-tumor samples were collected from 10 histopathologically and clinically diagnosed PTC patients at Tianjin Medical University General Hospital from January 2017 to March 2017. Total RNA was extracted using TRIzol reagent and the quality of the extracted RNA was checked by Agilent 2100 Bioanalyzer (Santa Clara, CA, USA). Then, the cDNA libraries were constructed and sequenced on a HiSeq 2000 at Beijing Genomics institution. The study was planned according to the ethical guidelines following the Declaration of Helsinki. This project was approved by the Ethics Committee of Tianjin Medical University General Hospital. All participants gave informed consent prior to enrollment in the investigation.

### UALCAN Database Analysis

UALCAN (http://ualcan.path.uab.edu) is a comprehensive, user-friendly, interactive web resource using TCGA RNA-seq data and the latest clinical data for 31 cancer types (23). It was designed to allow for the analysis of tumor and peri-tumor samples as well as different tumor types based on individual cancer stages, tumor grades, or other clinicopathological features and the relative gene expression of the subgroups. We selected key genes from the 13 hub genes to correlate to multiple clinical disease characteristics.

### Human Protein Atlas Analysis

The Human Protein Atlas (https://www.proteinatlas.org/) is a website containing immunohistochemistry (IHC) data to help investigate protein expression in human tissues and cells (24). Patient information, staining intensity, staining location, and sample number are available for each type of cancer. We assessed the expression of representative proteins in PTC and peri-tumor tissues of six key genes using IHC images after screening.

### Construction of a Prognostic Gene Signature

To assess the prognostic value of the hub genes, we downloaded the THCA clinical dataset from TCGA database and tested the six gene signature *via* multifactorial Cox regression analysis using the Sangerbox (http://sangerbox.com/Tool) online tool.

### DNA Methylation Interactive Visualization Database Analysis

DNA Methylation Interactive Visualization Database (DNMIVD); (http://119.3.41.228/dnmivd/index/) is a comprehensive annotation and interactive visualization database for DNA methylation profiles of diverse human cancers constructed with high throughput microarray data from TCGA and the GEO databases (25). To determine the best-fitting prognostic model, we established a multivariate proportional hazard regression model for the six hub genes. We utilized a Kaplan-Meier multivariate proportional hazards regression model to divide patients into high-risk and low-risk groups.

### Immune Infiltration Analysis

Tumor Immune Estimation Resource (TIMER; https://cistrome.shinyapps.io/timer/) is a web resource for systematic evaluation of the clinical impact of various immune cell populations on different cancer types, and consists of 10,897 samples from 32 cancer types. In this study, we analyzed the expression of six hub genes in THCA involved in tumor purity and the abundance of their immune infiltration (B cells, CD4+ T cells, CD8+ T cells, neutrophils, macrophages, and dendritic cells (DCs). Furthermore, we explored the relationship between gene copy number variation and the abundance of immune infiltration.

### Gene Set Enrichment Analysis

GSEA is the process of sorting genes by the degree of differential expression between two types of samples using a predefined set of genes, usually from functional annotations or results of previous experiments, and then verifying whether the predefined set of genes is enriched at the top or bottom of this sorting table. We downloaded the THCA gene expression matrix from TCGA database and performed GSEA analysis using the Sangerbox website (http://sangerbox.com/Tool) to predict potential hallmarks. A permutation test with 1,000 permutations was used to identify the significantly changed pathways. Thresholds of an adjusted p-value <0.05 were considered statistically significant.

### Statistical Analysis

Data were expressed as mean ± standard deviation. Analyses were done using GraphPad Prism (version 8.0; San Diego, CA, USA). Comparisons between the tumor and peri-tumor groups were performed using Student’s t-test. *P* value less than 0.05 were considered statistically significant.

## Results

### Screening of DEGs and Functional Enrichment Analysis

**Figure 1:**
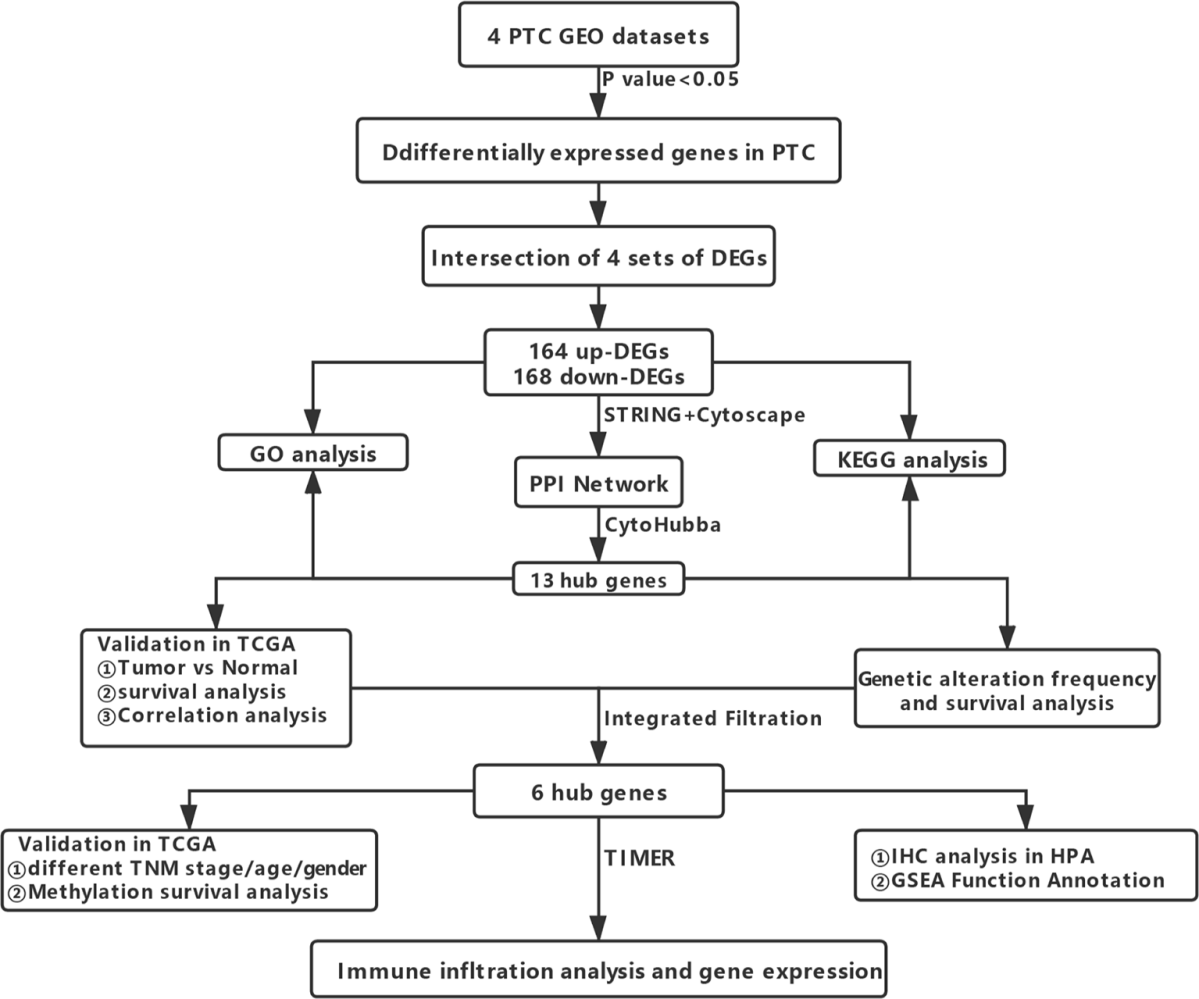
Flowchart showing the workflow for the identification, functional analysis, and verification of DEGs.

**Figure 2:**
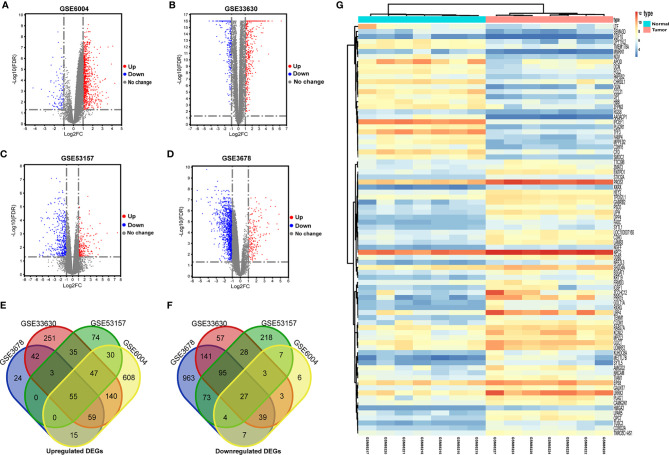
Identification of differentially expressed genes in PTC from public GEO datasets. **(A–D)** Volcano plots of DEGs in the four indicated datasets. DEGs, differentially expressed genes. X-axis: log_2_ (FC); Y-axis: -log_10_ (FDR) for each gene. Genes with FDR < 0.05 and FC > 1.0 or < -1.0 are considered DEGs for each dataset. Blue: downregulated genes; Grey: no differentially expressed genes; Red: upregulated genes. **(E, F)** Screening of DEGs in mRNA expression profiling datasets GSE3678, GSE6004, GSE33630, and GSE53157. **(E)** The overlapping regions indicate the commonly upregulated DEGs. **(F)** The overlapping regions indicate the commonly downregulated DEGs. **(G)** Representative heat map of the 82 overlapping genes between PTC and normal samples in the GSE3678 dataset. Orange represents higher expression and blue represents lower expression.

**Figure 3:**
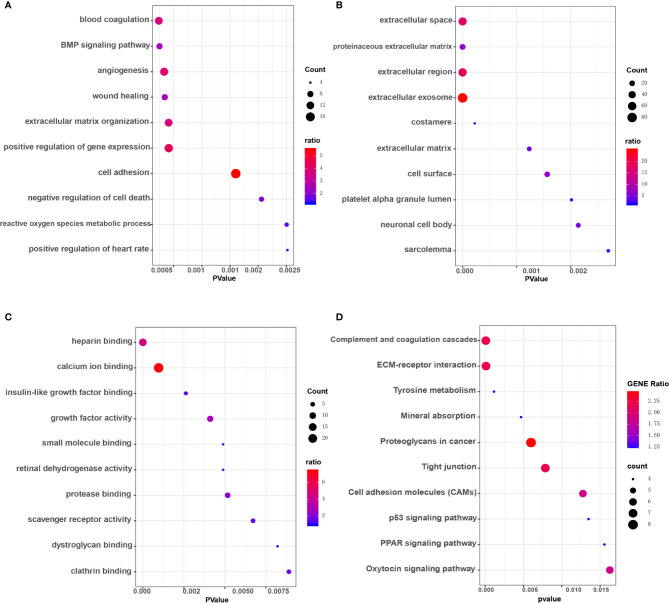
GO enrichment and KEGG pathway analysis of overlapping DEGs in at least three microarray datasets. **(A)** Biological processes (BP) enrichment analysis. **(B)** Cellular components (CC) enrichment analysis. **(C)** Molecular function (MF) enrichment analysis. **(D)** KEGG pathway analysis.

Workflows are presented for DEGs identification, function enrichment, and the validation analysis (**Figure 1**). To ensure more reliable quality of the datasets, GSE3678, GSE6004, GSE33630, and GSE53157 dataset deviations with those with higher levels were removed, leaving 76 PTC samples and peri-tumor samples for comparison. A total of 1,547, 1,050, 1,025, and 699 DEGs were identified for the GSE3678, GSE6004, GSE33630, and GSE53157 datasets, respectively (**Figures 2A–D**). Among the DEGs, 82 genes showed the same expression trend across the four datasets, including 55 upregulated genes (**Figure 2E**) and 27 downregulated genes (**Figure 2F**). Using the data profile GSE3678 as a reference, the heat map showed the distribution of significant differences among the 82 DEGs (**Figure 2G**). After filtering out 332 overlapping DEGs, 164 upregulated and 168 downregulated genes were discovered at the intersection of at least three microarray datasets for PTC and peri-tumor for GO and KEGG analyses. The data were visualized with Sangerbox software. The top 10 for each GO term and the top 10 KEGG pathways are listed. For BPs, DEGs were mainly enriched in blood coagulation, the bone morphogenetic protein signaling pathway, angiogenesis, wound healing, and extracellular matrix organization (**Figure 3A**). The CCs analysis revealed that DEGs were mostly enriched in the extracellular space, proteinaceous extracellular matrix, extracellular region, extracellular exosome, and costamere (**Figure 3B**). In molecular function, DEGs were primarily enriched for heparin binding, calcium ion binding, insulin-like growth factor binding, growth factor activity, and small molecule binding (**Figure 3C**). The KEGG pathway analysis indicated that the DEGs were predominantly enriched in the complement and coagulation cascades, extracellular matrix-receptor interaction, tyrosine metabolism, mineral absorption, proteoglycans in cancer, tight junction, cell adhesion molecules, p53 signaling pathway, PPAR signaling pathway, and oxytocin signaling pathway (**Figure 3D**).

### Key Genes Screened Through the PPI Network and Functional Enrichment Analysis

**Figure 4:**
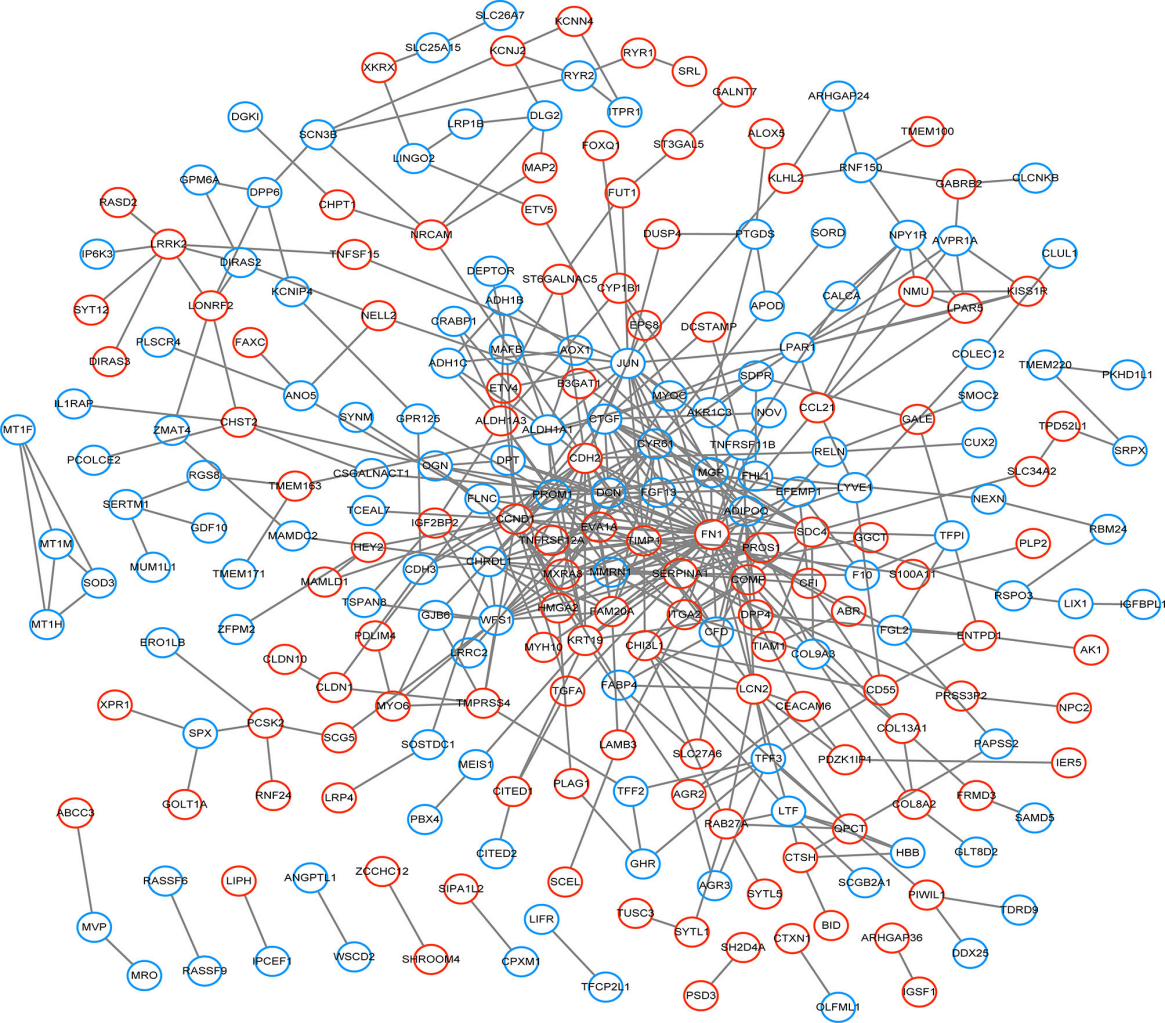
The PPI network of DEGs was constructed using Cytoscape. Red circles represent upregulated genes, while blue circles represent downregulated genes.

**Figure 5:**
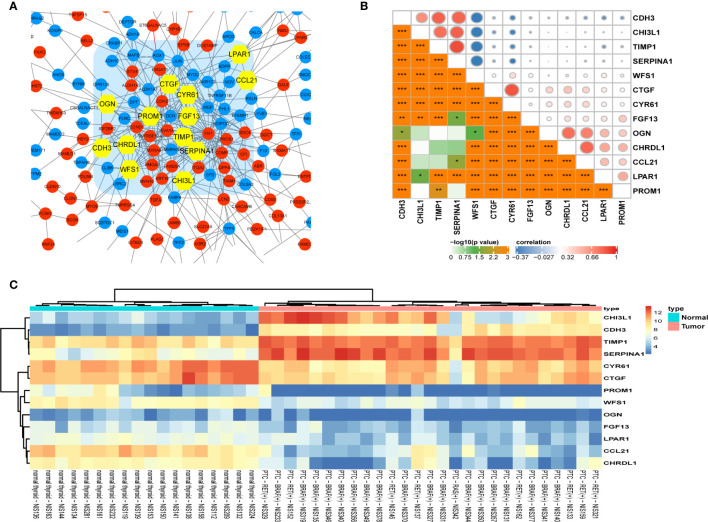
Identification and analysis of hub genes. **(A)** Thirteen statistically significant hub genes were screened using the Cytoscape software plugin cytoHubba. **(B)** Pearson’s correlation analysis of 13 hub genes. **(C)** The hierarchical clustering heat map of the 13 most significant hub genes was constructed using the GSE58545 dataset. Orange indicates that the expression of genes is relatively upregulated, blue indicates that the expression of genes is relatively downregulated. (*p< 0.05; **p < 0.01; ***p < 0.001).

**Figure 6:**
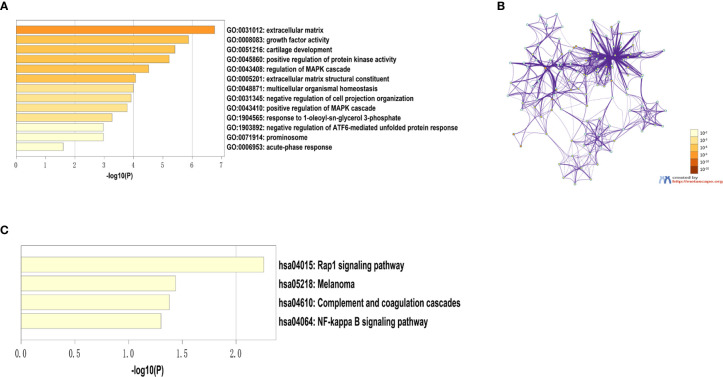
Results of the hub gene GO and KEGG pathway analysis. **(A)** Top 13 GO enrichment terms. **(B)** A network of GO-rich terms colored by p-value. **(C)** Top nine KEGG pathway enrichment terms.

332 DEGs previously screened were used in the PPI network analysis and a total of 242 DEGs (118 upregulated genes and 124 downregulated genes) were filtered into a PPI network containing 242 nodes and 466 edges using the STRING online database and were visualized using Cytoscape software (**Figure 4**). Thirteen hub genes were identified at the intersection of the top 65 DEGs calculated with 12 cytoHubba algorithms, including *CTGF, CDH3, CHRDL1, FGF13, LPAR1, OGN, TIMP1, CYR61, CHI3L1, SERPINA1, PROM1, CCL21*, and *WFS1* (**Figure 5A**). The correlation between the 13 hub genes were calculated using the TCGA-THCA dataset (**Figure 5B**). A Pearson’s correlation analysis revealed significant correlations among them. To validate these results, another GEO dataset (GSE58545) was used to measure the mRNA expression of the 13 hub genes. Rank clustering showed that the hub genes can distinguish PTC samples from peri-tumor group samples (**Figure 5C**). Furthermore, Metascape software was used for GO and KEGG pathway analysis. The top 13 GO enrichment terms(**Figures 6A, B**) and BPs included: cartilage development, positive regulation of protein kinase activity, regulation of MAPK cascade, multicellular organismal homeostasis, negative regulation of cell projection organization, positive regulation of MAPK cascade, response to 1-oleoyl-sn-glycer, negative regulation of ATF6-mediated unfolded protein response, acute-phase response. CCs include: extracellular matrix, and prominosome. Molecular function includes: growth factor activity, and extracellular matrix structural constituent. The top 4 KEGG pathways were Rap1 signaling, melanoma, complement and coagulation cascades, and the NF-kappa B signaling pathway (**Figure 6C**). Most of the pathways are involved in tumorigenesis and immune processes.

### Genetic Alterations of 13 Screened Hub Genes in PTC Patients

**Figure 7:**
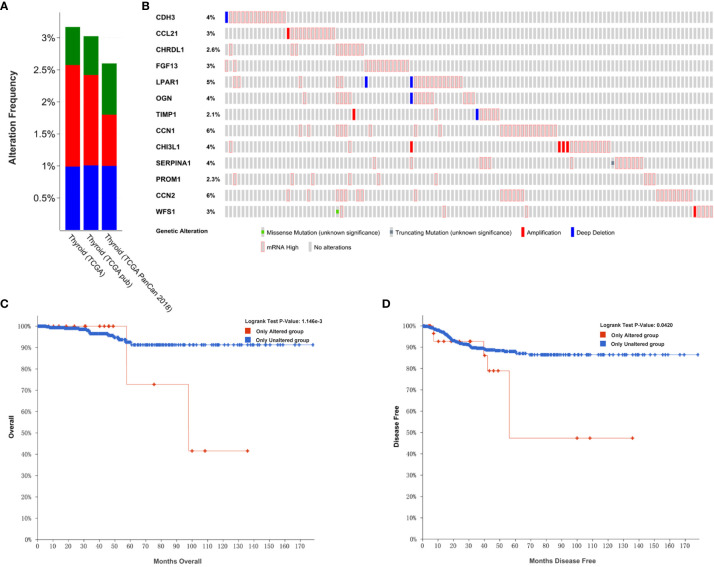
Genetic alterations linked to hub genes in THCA in the TCGA. **(A)** Thirteen hub gene alterations in THCA (TCGA, Firehose Legacy), PTC (TCGA, Cell 2014), THCA (TCGA, PanCancer Atlas). **(B)** Alteration frequencies of hub genes based on the PTC (TCGA, Cell 2014) dataset. **(C)** Kaplan-Meier plots comparing OS in cases with and without hub gene alterations. **(D)** Kaplan-Meier plots comparing DFS in cases with and without hub gene alterations.

In order to analyze the genetic alterations of the 13 hub genes in THCA in the cBioPortal online databases, the THCA (TCGA; Firehose Legacy), PTC (TCGA; Cell 2014), and THCA (TCGA, PanCancer Atlas) databases were used. Results showed that the percentages of genetic alterations in each dataset were 3.17% (16/505), 3.02% (15/496), 2.6% (13/500), respectively (**Figure 7A**). Specific to PTC, the alteration frequency of the 13 hub genes was 4% for *CDH3*, 3% for *CCL21*, 2.6% for *CHRDL1*, 3% for *FGF13*, 5% for *LPAR1*, 4% for *OGN*, 2.1% for *TIMP1*, 6% for *CYR61* (*CCN1*), 4% for *CHI3L1*, 4% for *SERPINA1*, 2.3% for *PROM1*, 6% for *CTGF* (*CCN2*), and 3% for *WFS1*. In addition, the predominant alterations were expression changes instead of mutations (**Figure 7B**).

We also performed an analysis of the correlation between cases with hub gene alterations and survival outcomes. Accordingly, results showed that cases with hub gene alterations were significantly associated with worse OS and DFS (p=1.146E-3; p=0.0420; **Figures 7C, D**).

**Figure 8:**
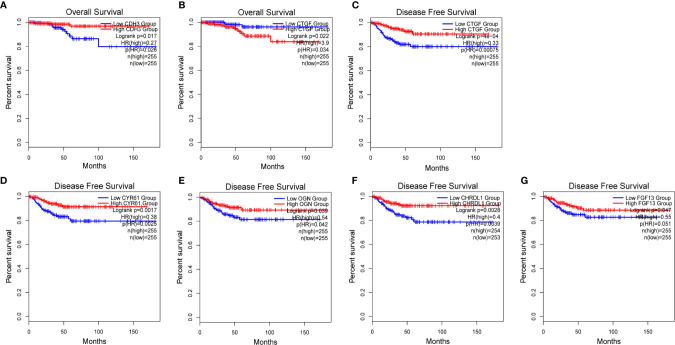
Survival analysis of 6 hub genes. **(A, B)** Kaplan-Meier survival analyses of TCGA PTC patients based on the expression of *CDH3* and *CTGF*. **(C–G)** Disease-free survival (DFS) of TCGA PTC patients based on *CTGF, CYR61, OGN*, *CHRDL1* and *FGF13*, respectively.

We used GEPIA to evaluate the contribution of the 13 hub genes to clinical outcomes. The results revealed that *CDH3* (p=0.017) was significantly associated with OS (**Figure 8A**). *CYR61* (p=0.0017), *OGN* (p=0.039), *CHRDL1* (p=0.0028), and *FGF13* (p=0.047) were significantly correlated with DFS (**Figures 8D–G**). Moreover, *CTGF* was involved in both OS (p=0.022) and DFS (p=0.0004; **Figures 8B, C**). The remaining hub gene survival analyses did not show any statistical significance (**Supplementary Figure 1**). In summary, we demonstrated that the 13 hub genes are involved in PTC, and six genes (*CDH3, CYR61, OGN, CHRDL1, FGF13*, and *CTGF*) were significantly correlated to patient outcomes.

### Validation of the Expression of Six Prognosis-Related Hub Genes

**Figure 9:**
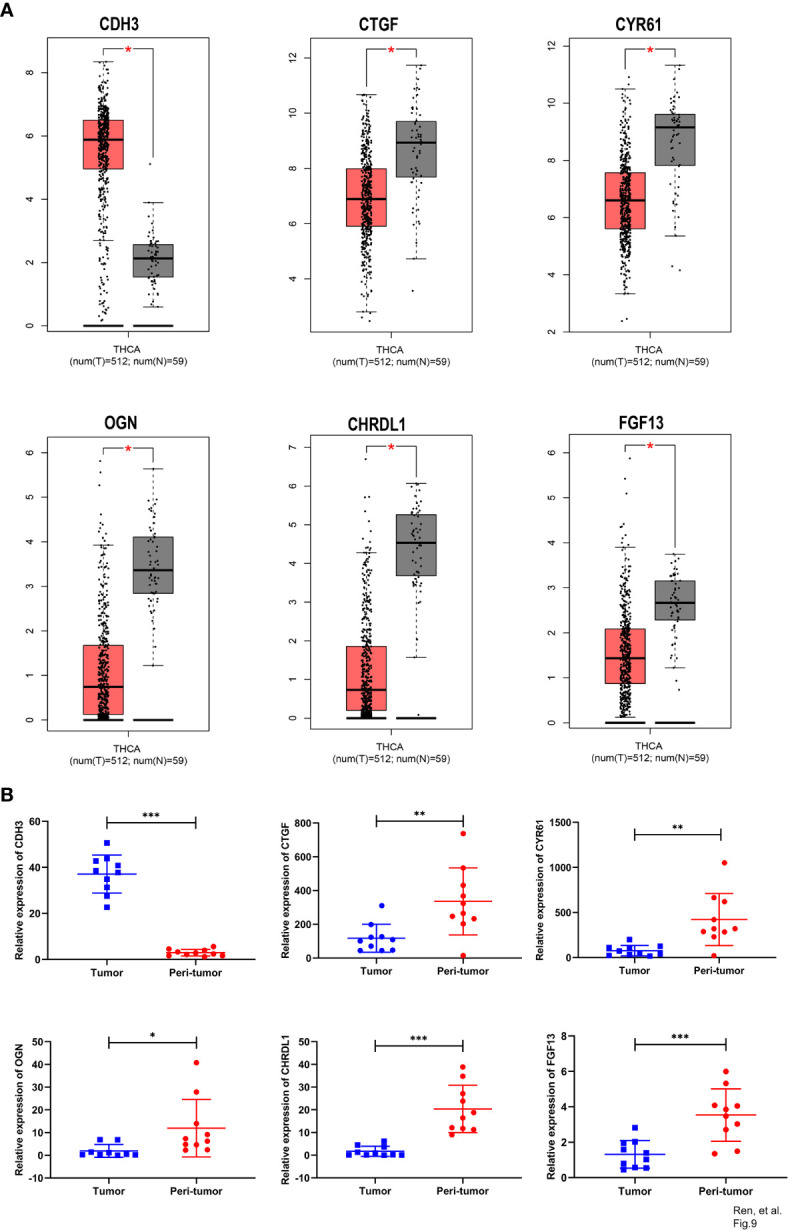
Comparison of expression of six hub genes between tumor and peri-tumor. **(A)** Box plots of *CDH3, CTGF, CYR61, OGN*, *CHRDL1* and *FGF13* expression in THCA tumor and peri-tumor tissues. **(B)** Scatter plots of *CDH3, CTGF, CYR61, OGN*, *CHRDL1* and *FGF13* were constructed using transcriptome sequence data of our own patients’ cohort. (*p<0.05; **p<0.01; ***p<0.001).

**Figure 10:**
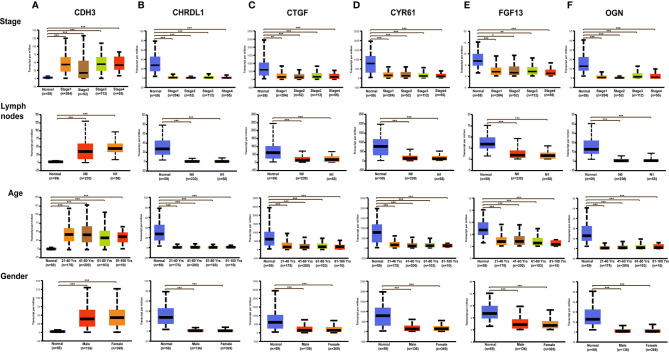
Expression of six hub genes in THCA subgroups stratified by clinical parameters in the UALCAN database. Boxplots showing the relative expression of six hub genes in normal individuals or THCA patients by tumor stage, lymph node metastases, gender, and age, respectively. **(A)** *CDH3*, **(B)** *CTGF*, **(C)** *CYR61*, **(D)** *OGN*, **(E)** *FGF13*, **(F)** *CHRDL1*. The center lines are the medians, and the bottom and top edges of the boxes are the 25th and 75th percentiles, respectively. ^*^p<0.05; ^**^p<0.01; ^***^p<0.00001.

**Figure 11:**
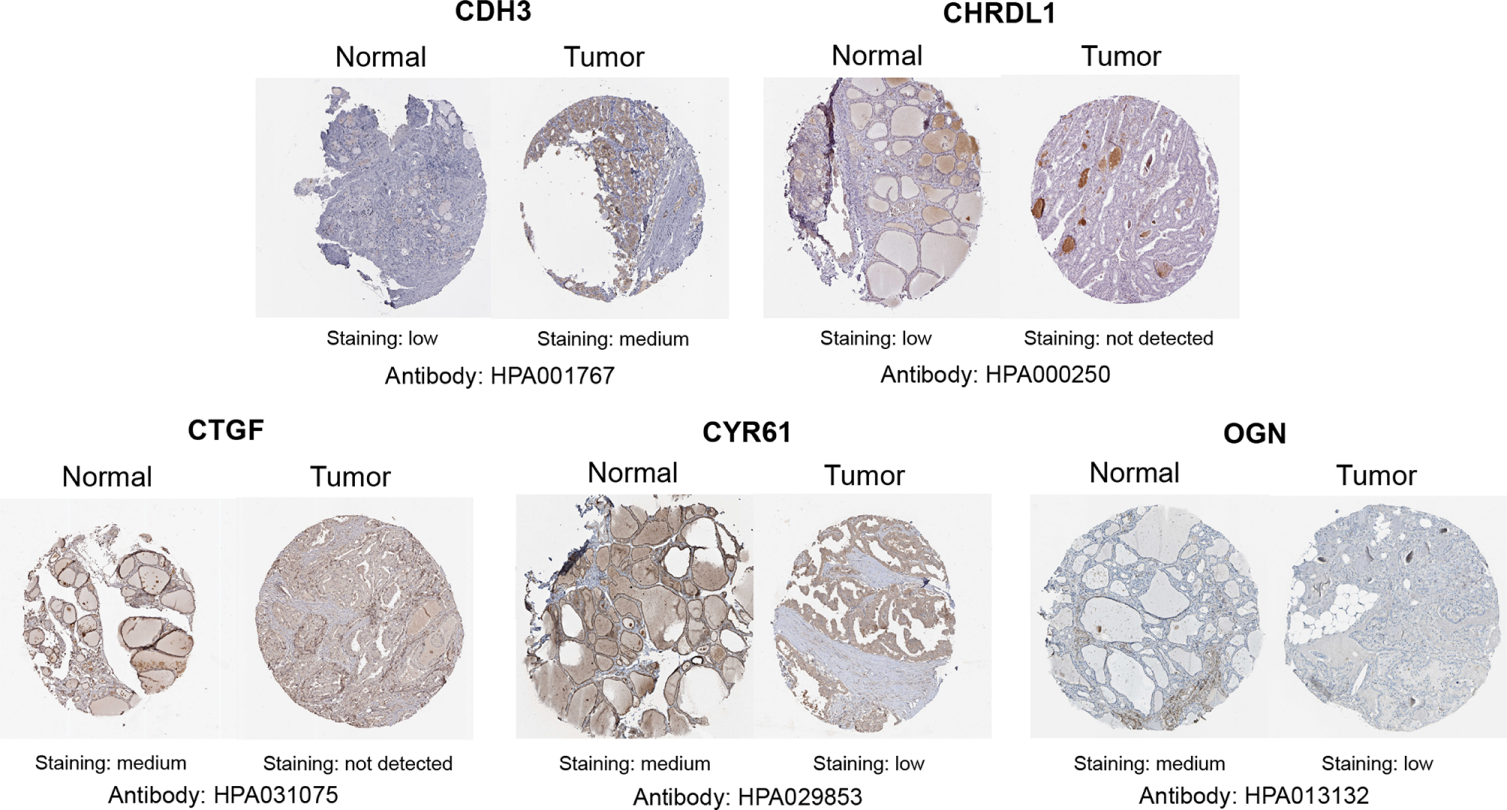
Representative immunohistochemistry staining of screened genes. Protein expression levels of CDH3, CHRDL1, CTGF, CYR61, and OGN in PTC tissue obtained from the Human Protein Atlas.

With GEPIA, we detected the transcript levels of six hub genes in 59 peri-tumor tissues and 512 THCA tissues from TCGA database. *CDH3* was found to be highly expressed, while *CTGF, CYR61, CHRDL1, OGN* and *FGF13* had significantly lower expression in tumor tissues compared with peri-tumor tissues (**Figure 9A**). In order to validate the bioinformatics analysis results, 10 paired PTC tumor and peri-tumor samples were collected and performed for transcriptome sequencing studies. Comparing with peri-tumor controls, the expression of *CTGF*, *CYR61*, *CHRDL1, OGN* and *FGF13* were significantly decreased, whereas *CDH3* significantly increased in PTC tissues (**Figure 9B**) which were consistent with the bioinformatics results obtained by TCGA dataset. Additionally, the expression of the six hub genes were analyzed according to various clinicopathological characteristics, which included tumor stage, lymph node metastases, sex, and age by using UALCAN software. *CDH3* transcript levels were significantly increased, while the expression of *CHRDL1, CTGF, CYR61, FGF13*, and *OGN* were significantly decreased in THCA patients compared with healthy subjects (**Figures 10A–F**). The expression levels of the six genes were not different when comparing tumor stages, lymph node metastasis, age, and sex (**Figures 10A–F**). To verify the transcriptome analysis results, immunohistochemistry data of the six genes were obtained from the Human Protein Atlas website. The protein expression levels of *CDH3, CHRDL1, CTGF, CYR61*, and *OGN* showed similar patterns of changes as the transcript levels (**Figure 11**).

**Figure 12:**
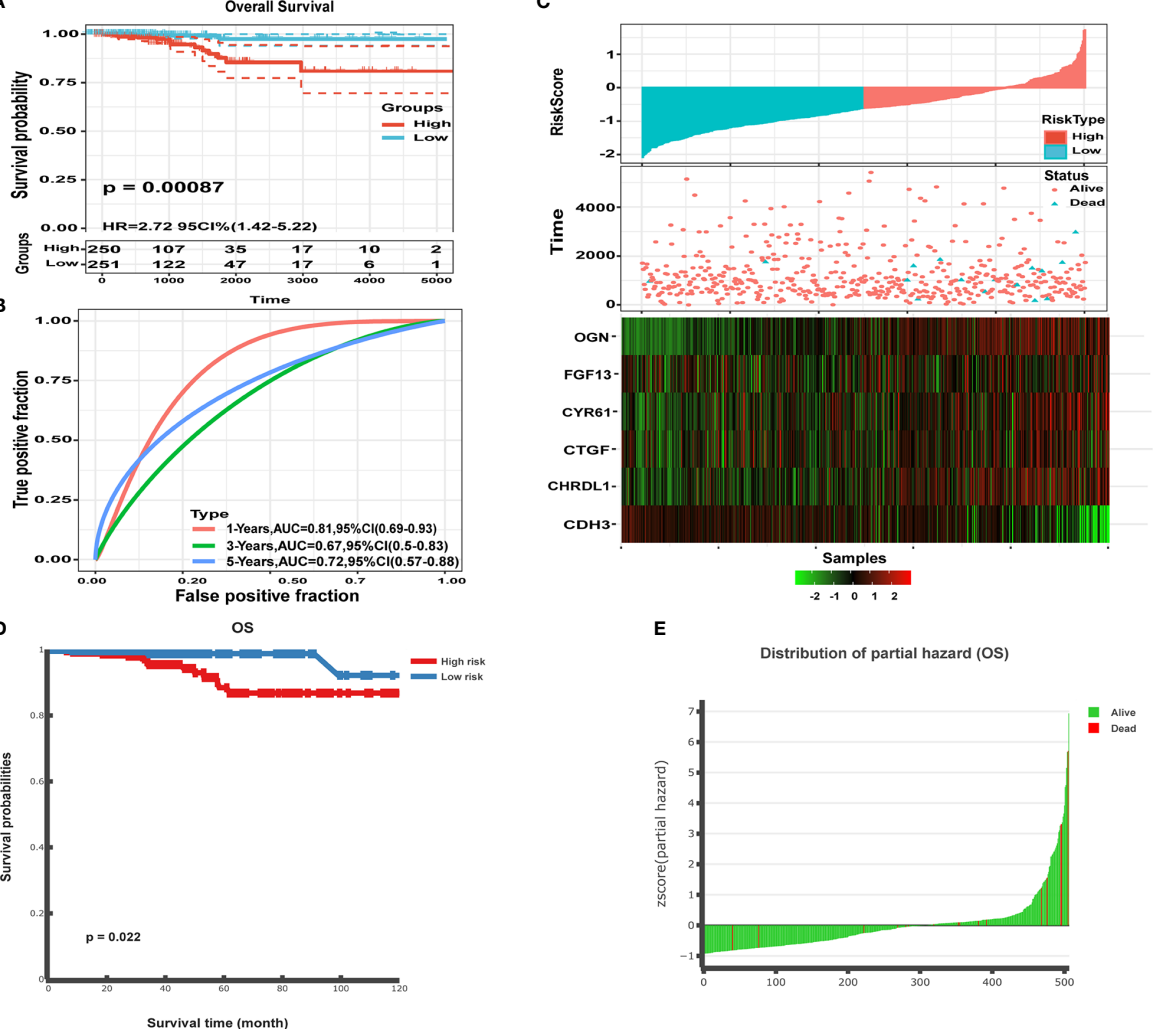
Performance of the six gene signature model. **(A)** Kaplan-Meier survival curve distribution of the six gene signature from the TCGA-THCA dataset. **(B)** ROC curve and AUC for the six gene signature classification. **(C)** TCGA-THCA set focused on risk score, survival time, survival status, and expression of the six gene signature. **(D)** Kaplan-Meier survival analysis for the patients in the THCA dataset. Patients were divided into low risk and high risk groups using the median cutoff value of the partial hazard. P-values were calculated using the log-rank test. **(E)** Z-score distribution of the prognostic classifier and patient survival status.

To validate the classification performance of the six gene signature model using different data platforms, we used TCGA data as an external dataset. We then calculated the risk score for each sample using the Sangerbox website and used the cut-off values of the training set to classify the samples into high and low risk groups. The low risk group had a significantly different prognosis than the high risk group (**Figure 12A**). An ROC analysis showed 1-, 3-, and 5-year AUCs of 0.81, 0.67, and 0.72 for the six gene signatures, respectively (**Figure 12B**). The data were presented for TCGA samples focusing on risk score, survival time, survival status, and expression of the six gene signature (**Figure 12C**). *CDH3* was highly expressed in tumor samples, whereas high expression of *CTGF, CYR61, OGN, FGF13*, and *CHRDL1* was associated with better prognosis and appeared to be a protective factor. Furthermore, we performed methylation survival analysis of the six gene signature using the DNMIVD tool. Patients were then classified into either high risk or low risk groups, using the median risk score as the cut-off point. Patients in the high risk group had a significantly shorter median OS than those in the low risk group (p<0.022; **Figure 12D**). We also calculated the Z-score distribution of the prognostic classifier and patient survival status (**Figure 12E**). Overall, our data showed that all six genes were significantly associated with THCA and could be used as a signature to sensitively and accurately determine prognosis in tumor patients.

### Relationship Between Key Gene Expression and Tumor Purity and Immune Infiltration

**Figure 13:**
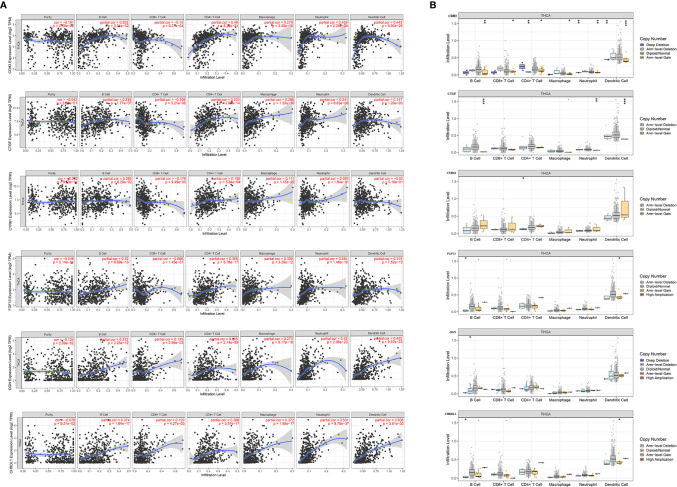
Correlation of expression of the six hub genes with immune infiltration in THCA. **(A)** Correlation of genes including *CDH3, CTGF, CYR61, FGF13, OGN*, and *CHRDL1* with tumor purity, and infiltration of B cells, CD8+ T cells, CD4+ T cells, macrophages, neutrophils, and dendritic cells. **(B)** The effect of copy number variation of *CDH3, CTGF, CYR61, FGF13, OGN*, and *CHRDL1* on the distribution of various immune cells. (*p < 0.05; **p < 0.01; ***p < 0.001).

The tumor microenvironment (TME) is composed of tumor cells, stromal cells, and infiltrating immune cells. The TIMER database was utilized to analyze the association of specific gene expression levels with THCA immune populations. Interestingly, *CDH3* (r=-0.101, p=2.59E-02) and *OGN* (r=-0.104, p=2.09E-02) was negatively correlated with tumor purity. Moreover, the expression of *CDH3, CTGF, OGN*, and *CHRDL1* was significantly correlated with the infiltration of B cells, CD8+ T cells, CD4+ T cells, macrophages, neutrophils, and DCs. The expression of *CYR61* was significantly correlated with the infiltration of CD8+ T cells, CD4^+^ T cells, and neutrophils. The expression of *FGF13* was significantly correlated with the infiltration of B cells, CD4+ T cells, macrophages, neutrophils and DCs (**Figure 13A**). Interestingly, the copy number variation (CNV) of *FGF13* and *CHRDL1* were significantly correlated with infiltration levels of B cells and DCs. *OGN* was associated with B cells and *CYR61* was associated with CD4+ T cells. *CTGF* and *CDH3* were both associated with B cells, neutrophils, and DCs, and the CNV of *CDH3* was also significantly correlated with infiltration levels of CD8+ T cells, CD4+ T cells, and macrophages (**Figure 13B**).

### Significant Pathways and Genes Obtained by GSEA

**Figure 14:**
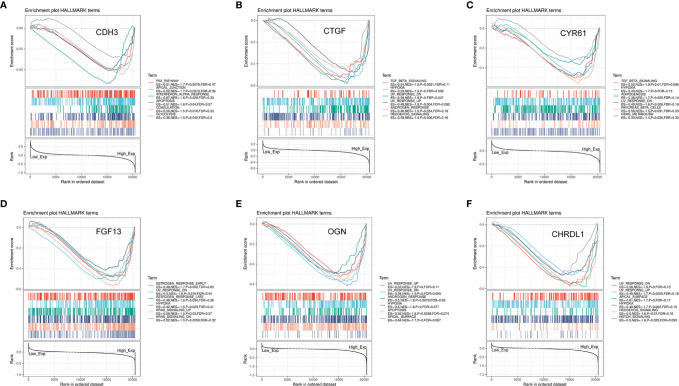
Significant genes associated with six hub genes and hallmark pathways in THCA obtained by GSEA. **(A–F)** Top six gene sets according to a GSEA enrichment score for *CDH3, CTGF, CYR61, FGF13, OGN*, and *CHRDL1*.

To further explore the potential function of the six key genes in THCA, we performed GSEA on TCGA-THCA RNA-seq data. A total of 100 significant genes were acquired by GSEA with both positive and negative correlations. GSEA was used to conduct a hallmark analysis for *CDH3, CTGF, CYR61, FGF13, OGN*, and *CHRDL1*. *FGF13, CTGF, CHRDL1, OGN*, and *CYR61* were all enriched through hypoxia and UV response downregulation pathways. Meanwhile, *CTGF, CHRDL1*, and *OGN* were all enriched in UV response upregulation pathways. The five genes (*FGF13, CTGF, CHRDL1, OGN*, and *CYR61*) were also enriched through pathways such as TGF-β signaling, adipogenesis, hedgehog signaling, androgen response, apical surface, apoptosis, notch signaling, KRAS signaling upregulated and down regulated genes, and early and late estrogen response. The signaling pathways most involved with *CDH3* include the P53 pathway, apical junction, apoptosis, interferon-alpha response, coagulation, and glycolysis (**Figures 14A–F**). Some of these pathways are associated with tumor development. Remarkably, these pathways were significantly enriched in high risk samples.

## Discussion

In recent years, the development of bioinformatics has allowed for the discovery of new biomarkers for PTC with extensive research using microarrays and RNA-seq (10). In our present study, we analyzed four microarray datasets, including 76 PTC and 55 peri-tumor samples. We filtered 332 overlapping DEGs in at least three microarray datasets. GO terminology and KEGG pathway analysis showed that the screened DEGs are implicated in the pathogenesis of PTC. A correlated PPI network containing 242 nodes and 466 edges was constructed and visualized utilizing the STRING database and Cytoscape software. The 13 most significant hub genes were screened using the cytoHubba algorithm with Cytoscape software and were identified using cBioPortal. Gene alterations of the 13 hub genes occurred in 243 (49%) of the queried samples. Almost every hub gene had different kinds of genetic alterations, and we found that mRNA upregulation, amplification, and deep deletions were the three most commonly occurring types of aberrations. Interestingly, we found the cases with hub gene alterations correlated with worse OS (p=1.146e^-3^) and DFS (p=0.0420). The results showed that the cases in which the hub gene was altered predicted poor OS and DFS. Furthermore, the KEGG results suggest that most of the 13 hub gene-related pathways are mainly involved in tumorigenesis. According to previous studies, Rap1 activation is involved in RET/PTC1-mediated BRAF kinase and p42/p44 mitogen-activated protein kinase stimulation, which may contribute to the transformed phenotype of RET/PTC-expressing thyroid cells and tumorigenesis (26). Complement and coagulation cascades can eliminate pathogens and damaged host cells, and when their activation is uncontrolled or excessively activated tumorigenesis may be enhanced (27, 28). KLF5 promotes the tumorigenesis and metastasis potential of THCA cells through the NF-κB signaling pathway, which enhances proliferation, invasion, and migration of cadmium-induced GPER-positive THCA cells (29, 30). These results shed some light on the role of the hub genes and their related signaling pathways in the occurrence and development of PTC. Similarly, GO enrichment analysis showed that the 13 hub genes were mainly related to tumor development, especially the ATF6 and MAPK pathways. According to published literature, ATF6 mediates apoptosis by inducing unfolded proteins to reduce myeloid leukemia sequence 1 (Mcl-1) proteins (31). In our analysis, hub genes were enriched in negatively regulating ATF6-mediated unfolded proteins and increased Mcl-1 expression, which could lead to malignant cell growth and apoptotic escape (32). The MAPK signaling cascade regulates important cellular processes, including cell proliferation, gene expression, cell survival, death, and cell motility in mammals. It has been shown that TNFR1 and CD95 can induce anti-apoptotic signals through the MAPK proliferative cascade response, which can contribute to malignant tumor cell metastasis and resistance to anticancer drugs (33, 34). Until now, THCA has been regarded as a largely MAPK-driven cancer, with approximately 70% of THCAs being associated with activation of the mutations in this pathway (35). Therefore, it is reasonable to assume that the 13 screened hub genes might play a pivotal role in the development of PTC.

To further evaluate the reliability of hub genes and identify genes that can be used as PTC biomarkers, we conducted a gene expression and Kaplan-Meier analysis of 13 hub genes. Six genes came out of this analysis: *CDH3, CTGF, CYR61, FGF13, CHRDL1*, and *OGN*. P-cadherin/*CDH3* belongs to the family of classical calcium adhesion proteins, which are cell adhesion molecules involved in cellular localization and tissue integrity, and has been implicated in many types of cancers, such as hepatocellular carcinoma (36), pancreatic cancer (37), and breast cancer (38). However, the role of P-cadherin in THCA remains unclear. *CTGF* is a stromal cell multifunctional protein that regulates cell migration, proliferation, and differentiation, as well as several neural-related processes like stimulation of nerve function, glial bridging, and natural spinal cord regeneration (39). *CTGF* has been widely reported as an independent prognostic marker for colorectal cancer metastasis (40). However, our knowledge of the role of *CTGF* in PTC development are little. *CYR61* is a stromal cellular protein located in the extracellular matrix and is associated with wound healing, angiogenesis, and osteoblast differentiation. *CYR61* is highly expressed in peri-tumor thyroid tissues, so it is necessary to further explore the relationship between *CYR61* expression and the molecular mechanisms of tumorigenesis. On the other hand, the prognostic roles of *FGF13, CHRDL1*, and *OGN* in PTC are still uncertain. The transcription levels of *CTGF, CYR61*, *FGF13, CHRDL1*, and *OGN* were significantly higher in health controls than in THCA patients in the subgroup analyses involving disease stage, lymph node metastasis, gender, and age. Decreased gene expression was associated with poorer survival, suggesting that these five hub genes were tumor suppressor genes. Whereas *CDH3* was significantly upregulated in THCA tissues and positively correlated with tumor stage, lymph node metastasis, sex, and age which indicated that *CDH3* was an oncogene. We also referred to DNMIVD to explore the relationship between methylation status and survival curves of the designated hub genes in THCA. We found that the median OS of patients in the high risk group was significantly shorter than that of patients in the low risk group, which may suggest that methylation of the six hub genes is associated with poor prognosis in PTC.

Gene signatures are currently being used in clinical practice, and gene expression profiling to screen for new cancer prognostic markers is now a promising high-throughput molecular identification method. In this study, we validated the classification performance of the six gene signature model by TCGA platform data due to the fact that the survival time were not available in the GEO database. The ROC curves showed that our six gene signature had a high AUC, implying that all six genes can be used as biomarkers to sensitively and accurately predict prognosis.

To further explore the biological functions of the six hub genes, we performed a tumor immune infiltration analysis with TIMER and GSEA for each hub gene. Our results revealed that expression of each hub gene correlated with most infiltrating immune cells including B cells, CD4+ T cells, CD8+ T cells, neutrophils, macrophages, and dendritic cells (DCs) in THCA tissue specimens. Also, *CDH3* and *OGN* expression were positively correlated with tumor purity. Moreover, GSEA results indicated that the six hub genes mainly enriched in hypoxia, the TGF-β signaling pathway, UV response pathways, and notch signaling. Hypoxia is one of the features of solid tumors that directly contributes to the malignant properties of cancer, including tumor invasion, progression, and metastasis (41, 42). Hypoxia also is a distinctive feature of the TME. The TME included non-tumor cells that existed in and around the tumor, and had a significant impact on the genomic analysis of tumor samples (43). Hypoxic signals increase resistance to T cell-mediated killing by enhancing CTLA-4 expression on CD8+ T cells, as well as recruiting Tregs, myeloid-derived suppressor cells, and tumor associated macrophages (TAMs) into the TME (44). Therefore, exploration of hypoxia and TME associated biomarkers may be potentially valuable for the detection and treatment of cancer. In addition, TGF-β pathway can influence tumor progression through regulating immune cells in the TME. One of the most notable functions of TGF-β is the induction of M2 macrophage polarization. TGF-β-rich TMEs may promote tumor immune evasion by suppressing the inflammatory function of macrophages, DCs maturation and neutrophils differentiation (45–47). On the other hand, the TGF-β signaling pathway is critical in promoting CD4+ T cells differentiated into Tregs which enhance tolerance to tumor antigens and promote immune evasion (45, 48). Deep understanding of TGF-β signal pathway in TME would throw lights on the interaction of immune cells and tumor progression. Similarly, notch signaling and UV response pathways also involved in tumor progression through affecting the immune cells in the TME (49, 50).

In the present study, we screened six hub genes that are associated with immune infiltration and are enriched in the above GSEA pathway. The six gene signature could predict the prognosis of THCA patients. Additional work is needed to further explore the interaction between our screened hub genes and immune cell infiltration in the TME.

## Conclusion

Taken above, our study identified a six genes signature by using multiple cohort datasets and comprehensive bioinformatics analyses. The six genes were enriched in immune-related pathways which closely related to tumorigenesis and prognosis of PTC. However, more samples from different databases and more experimental studies still be needed to validate our observations.

## Data Availability Statement

Publicly available datasets were analyzed in this study. These data can be found here: GEO (http://www.ncbi.nlm.nih.gov/geo/) and TCGA (https://portal.gdc.cancer.gov/).

## Ethics Statement

The studies involving human participants were reviewed and approved by the Medical Ethics Committee of Tianjin Medical University General Hospital, and informed consent of all participants was obtained.

## Author Contributions

HR and NZ designed the study and performed data analysis. HR and XL revised the images. HR and FL performed the literature search and data collection. NZ and XH revised the manuscript. All authors contributed to the article and approved the submitted version.

## Funding

This work was supported by the National Natural Science Foundation of China (Grant No. 81672641).

## Conflict of Interest

The authors declare that the research was conducted in the absence of any commercial or financial relationships that could be construed as a potential conflict of interest.

## Supplementary Material

Supplementary Figure 1 Survival analysis of the remaining hub genes. **(A–K)** Overall Survival (OS) analysis of the 11 hub genes: *CCL21, FGF13, CHI3L1, LPAR1, OGN, PROM1, SERPINA1, WFS1, CHRDL1, CYR61*, and *TIMP1*. p < 0.05 was regarded as statistically significant. **(L–S)** Disease-Free Survival (DFS) analysis of eight hub genes: *CCL21, CDH3, CHI3L1, TIMP1, LPAR1, PROM1, SERPINA1*, and *WFS1*. p < 0.05 was regarded as statistically significant.

## Abbreviations

GEO, Gene Expression Omnibus ; PTC, Papillary thyroid cancer ; DTC, differentiated thyroid cancer; DEGs, differentially expressed genes; THCA, Thyroid Cancer; GO, Gene Ontology; KEGG, Kyoto Encyclopedia of Genes and Genomes; BP, biological processes; CC, cellular components; MF, molecular functions; PPI, protein-protein interaction; TCGA, The Cancer Genome Atlas; TNM, Tumor Node Metastasis; OS, overall survival; DFS, disease-free survival; GSEA, Gene Set Enrichment Analysis; HPA, Human Protein Atlas; TME, the tumor microenvironment; TAMs, tumor associated macrophages.
