# Supplementary figures and images for: Identification of a Six Gene Prognosis Signature for Papillary Thyroid Cancer Using Multi-Omics Methods and Bioinformatics Analysis
Source: Front Oncol. 2021 Mar 18;11:624421. doi: 10.3389/fonc.2021.624421 (PMC8012734; doi:10.3389/fonc.2021.624421)

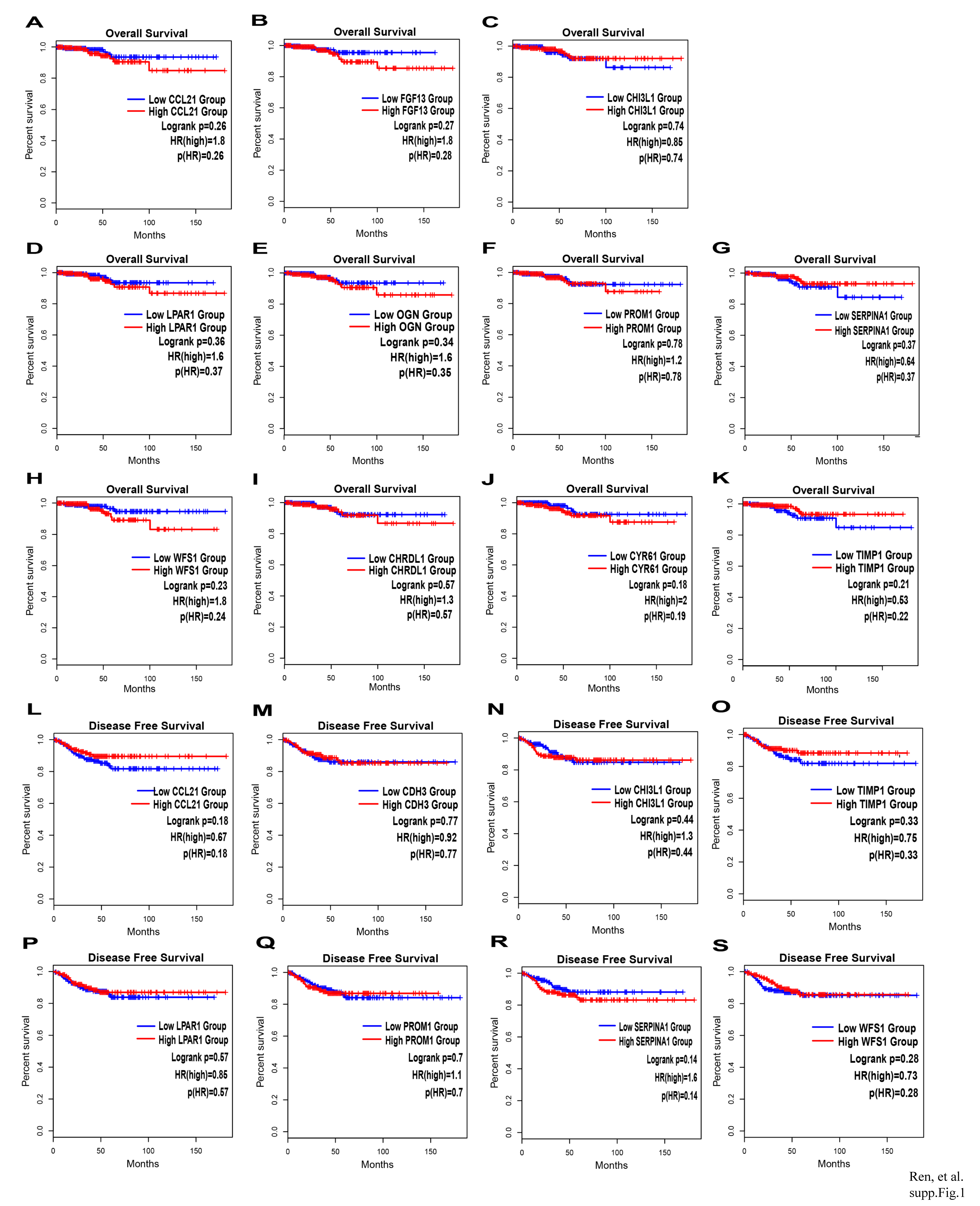

Supplement: Supplementary Figure 1 — Survival analysis of the remaining hub genes. (A–K) Overall Survival (OS) analysis of the 11 hub genes: CCL21, FGF13, CHI3L1, LPAR1, OGN, PROM1, SERPINA1, WFS1, CHRDL1, CYR61, and TIMP1. p < 0.05 was regarded as statistically significant. (L–S) Disease-Free Survival (DFS) analysis of eight hub genes: CCL21, CDH3, CHI3L1, TIMP1, LPAR1, PROM1, SERPINA1, and WFS1. p < 0.05 was regarded as statistically significant. [file Image_1.tif]
